# Hmgb1 Silencing in the Amygdala Inhibits Pain-Related Behaviors in a Rat Model of Neuropathic Pain

**DOI:** 10.3390/ijms241511944

**Published:** 2023-07-26

**Authors:** Peyton Presto, Guangchen Ji, Olga Ponomareva, Igor Ponomarev, Volker Neugebauer

**Affiliations:** 1 Department of Pharmacology and Neuroscience, Texas Tech University Health Sciences Center, Lubbock, TX 79430, USA; 2 Center of Excellence for Translational Neuroscience and Therapeutics, Texas Tech University Health Sciences Center, Lubbock, TX 79430, USA; 3 Garrison Institute on Aging, Texas Tech University Health Sciences Center, Lubbock, TX 79430, USA

**Keywords:** amygdala, neuropathic pain, Hmgb1, neuroimmune signaling, behavior

## Abstract

Chronic pain presents a therapeutic challenge due to the highly complex interplay of sensory, emotional-affective and cognitive factors. The mechanisms of the transition from acute to chronic pain are not well understood. We hypothesized that neuroimmune mechanisms in the amygdala, a brain region involved in the emotional-affective component of pain and pain modulation, play an important role through high motility group box 1 (Hmgb1), a pro-inflammatory molecule that has been linked to neuroimmune signaling in spinal nociception. Transcriptomic analysis revealed an upregulation of Hmgb1 mRNA in the right but not left central nucleus of the amygdala (CeA) at the chronic stage of a spinal nerve ligation (SNL) rat model of neuropathic pain. Hmgb1 silencing with a stereotaxic injection of siRNA for Hmgb1 into the right CeA of adult male and female rats 1 week after (post-treatment), but not 2 weeks before (pre-treatment) SNL induction decreased mechanical hypersensitivity and emotional-affective responses, but not anxiety-like behaviors, measured 4 weeks after SNL. Immunohistochemical data suggest that neurons are a major source of Hmgb1 in the CeA. Therefore, Hmgb1 in the amygdala may contribute to the transition from acute to chronic neuropathic pain, and the inhibition of Hmgb1 at a subacute time point can mitigate neuropathic pain.

## 1. Introduction

Chronic pain is a pervasive healthcare issue, affecting approximately 20% of the global population each year [1,2,3]. The experience of pain is a complex phenomenon due to an intricate interplay between sensory, emotional-affective and cognitive components. Elaborate relationships between these dimensions are particularly challenging to the development of effective therapeutic strategies for chronic pain relief; the majority of current treatment options are associated with unwanted side effects and have variable efficacy [4,5]. Difficulties in the identification of successful solutions are partially attributed to a lack of complete understanding regarding mechanisms and targets that are involved in the development and maintenance of a chronic pain state. Though maladaptive neuroplasticity has generally been regarded to play a critical contribution in the transition from acute to chronic pain [6], the mechanistic investigation of specific synaptic and cellular changes that can drive this evolution remains a prominent area of pain research. Recently, a new line of focus has shifted to the role of glial cells in pain processing, as they are the key components of the central nervous system and can release cytokines and chemokines that act as powerful neuromodulators. Complex interactions with neurons may contribute to synaptic modulation and neuroplasticity, as well as exhibit a pronounced influence over pain-related behaviors [7]. Therefore, the development of novel therapeutic approaches that target neuroimmune signaling mechanisms may represent a promising avenue for chronic pain resolution. However, neuroimmune signaling in the brain is an understudied area of pain research despite evidence from clinical studies [8,9].

The amygdala is a limbic brain structure located in the medial temporal lobe that has been well established as a critical player in the emotional-affective component of pain and pain modulation [10,11,12,13,14,15,16,17]. The amygdala circuitry conveys emotional significance to multimodal sensory information in pain states [17,18,19]. Amygdala output connects to descending pain modulatory systems, as well as other brain circuits that are implicated in behaviors, and emotional and cognitive processing [19,20,21,22,23,24]. The amygdala is composed of functionally different nuclei and subdivisions, with the central nucleus of the amygdala (CeA) serving as a major output center [12,19,25,26]. Neuroplasticity within the amygdala has been associated with pain-related behaviors under various pain conditions [12,15,18,27,28], rendering the amygdala a hub for emotional-affective and nociceptive integration and influence on pain behaviors and perception. Therefore, the amygdala is a desirable region of focus in the development of therapeutics for pain treatment, but mechanisms of the transition from acute to chronic pain are not clear.

High motility group box 1 (Hmgb1) is a highly conserved nuclear DNA-binding protein that has been implicated as a critical component of diverse physiological and pathological processes [29,30,31,32]. Hmgb1 is an important transcriptional regulator that plays a key role in autophagy and metabolism; it is secreted into the extracellular space in response to various stimuli, where it serves as a danger-associated molecular pattern to induce sterile inflammation and cell migration [33,34,35]. Recently, Hmgb1 has also been demonstrated to be a prominent mediator of neuroinflammation and neurodegeneration, predominantly through neuroimmune interactions. Evidence suggests a role of Hmgb1 in peripheral and spinal nociception, including in neuropathic pain conditions [36,37,38]. Hmgb1 can act on microglia by binding to toll-like receptors 2 and 4 (TLR2 and TLR4) and the receptor for advanced glycation end products (RAGE) [39,40] to trigger microglial activation, intracellular signaling cascades involving nuclear factor kappa B (NF-κB), and the release of pro-inflammatory cytokines, which can incite neuroinflammation and neuronal damage. Alternatively, Hmgb1 can also act on neurons to trigger neuronal hyperexcitability and chronic pain persistence [41,42,43]. However, the role of Hmgb1 in the brain mechanisms of neuropathic pain, particularly its interactions with amygdala neurons and glial cells in pain-related neuroplasticity and pain modulation, is largely unknown. Therefore, Hmgb1 represents an intriguing avenue of exploration in the development of novel therapeutics for chronic neuropathic pain relief.

The purpose of this study was to examine the role of Hmgb1 in neuropathic pain-related amygdala function and behaviors with regard to the temporal aspects of neuropathic pain development in male and female rats. Here we characterize the mRNA expression profiles of Hmgb1 in the right and left CeA at the acute and chronic stages of a spinal nerve ligation (SNL) rat model of neuropathic pain, as previous evidence has suggested right-hemispheric lateralization of pain processing in the amygdala. Next, we tested the behavioral consequences of Hmgb1 knockdown with small interfering RNA (siRNA) given as a pre-treatment 2 weeks before or post-treatment 1 week after SNL induction. Our findings point to a previously unexplored temporal and hemisphere-specific role of Hmgb1 in neuropathic pain-related processing within the CeA and provide support for the further investigation of pain-related neuroimmune signaling mechanisms in the amygdala.

## 2. Results

Previous evidence suggests that Hmgb1 may play a critical role in neuropathic pain pathogenesis through neuroimmune interactions in the periphery and spinal cord [36,37,38], but its contribution to the brain mechanisms of pain remains to be determined, particularly between the sexes. In this study, we investigated the expression of Hmgb1 in the amygdala and its role in neuropathic pain-related behaviors in male and female rats. Specifically, we tested the hypothesis that Hmgb1 is a critical transcription factor that contributes to pain-related processing within the amygdala, and that the inhibition of this signaling within the amygdala can reduce pain-related behaviors in both sexes.

### 2.1. Expression Levels of Hmgb1 in the Left and Right CeA in Neuropathic Pain

We first sought to identify individual genes, biological and functional groups, and molecular pathways that were affected by chronic neuropathic pain (4-week SNL model, see Section 4.2 “Neuropathic pain model”) within the left and right CeA in male rats using bulk RNA sequencing (see Section 4.4 “Gene expression using bulk RNA sequencing”). The right–left comparison was performed due to previous evidence for right-hemispheric lateralization of pain processing in the amygdala [14,44,45]. Of the DEGs that reached the statistical threshold of 5% FDR, those that have previously been implicated in neuroimmune signaling were selected for further investigation as potential therapeutic targets for chronic neuropathic pain. Hmgb1 was initially included in this cohort of candidate DEGs, as it has been shown to be a mediator of neuroimmune responses in many divergent clinical conditions including sepsis, atherosclerosis, cancer, and alcohol use disorder, as well as chronic pain at the peripheral and spinal levels [37,46,47,48,49]. The comprehensive results of this analysis will be presented in an upcoming publication, but for the purposes of this study we have elected to highlight Hmgb1 as a target of interest due to its link to neuroimmune signaling and peripheral and spinal pain mechanisms. Our data presented here also provide evidence for an important role of Hmgb1 in the amygdala in pain-related behaviors (see Section 2.3 and Section 2.4).

**Figure 1:**
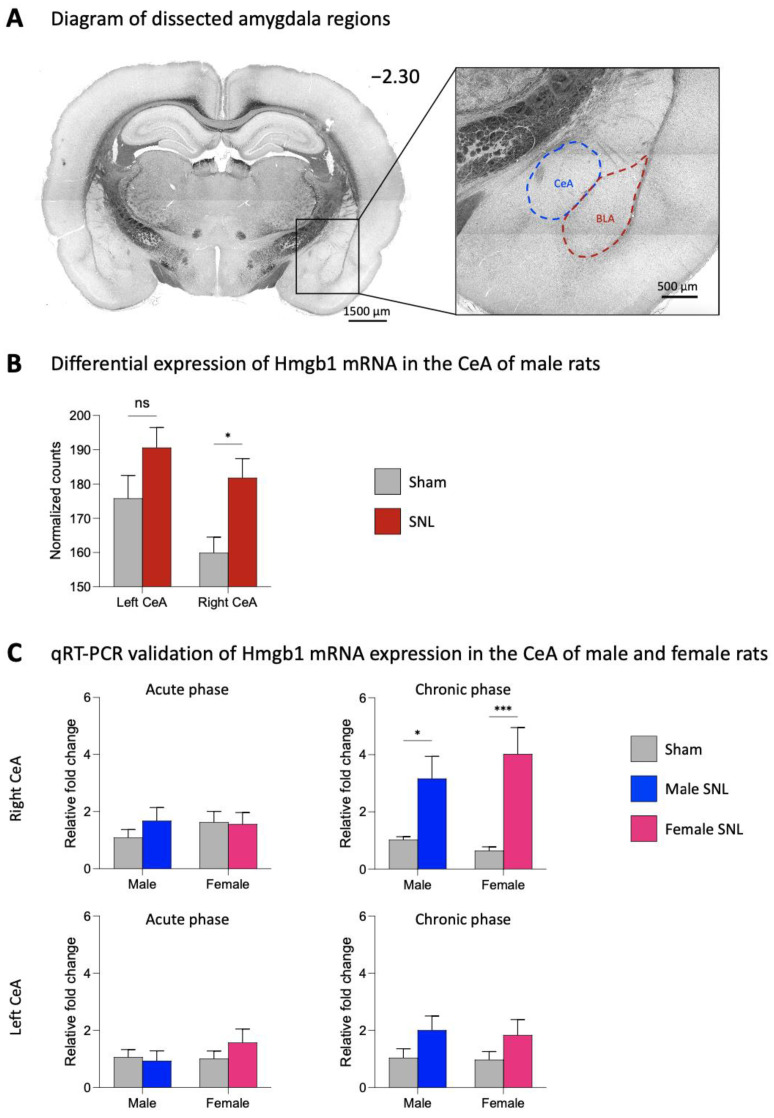
mRNA expression levels of Hmgb1 in the spinal nerve ligation (SNL) model of neuropathic pain. (**A**) Location of the dissected amygdala regions (CeA, blue; BLA, red) in a coronal brain slice. Numbers indicate distance from the bregma. (**B**) At the chronic (4 week) phase of the SNL model, brains were extracted from male rats and the left and right CeA were dissected out for bulk RNA sequencing. Differential expression analysis revealed an upregulation of Hmgb1 in the right but not the left CeA of male SNL rats (*n* = 6) compared to sham control (*n* = 6). * *p* < 0.05, ns, not significant, two-way ANOVA with Šidák’s post hoc tests, compared to the same-hemisphere sham. (**C**) At both the acute (1 week) and chronic (4 week) phases of the SNL model, brains were extracted from male and female rats, and the left and right CeA were dissected out for mRNA validation. qRT-PCR analysis of mRNA expression levels for Hmgb1 was performed on left and right CeA tissues at both time points. Data were normalized to the male sham group for both the acute and chronic phases. Hmgb1 mRNA expression in the chronic phase was upregulated in the right but not the left CeA in both male (*n* = 6) and female (*n* = 6) SNL rats compared to the sham control (male, *n* = 9; female, *n* = 7). In the acute phase, no significant differences in Hmgb1 mRNA expression were seen in the left or the right CeA for either sex. *, *** *p* < 0.05, 0.001, two-way ANOVA with Šidák’s post hoc tests, compared to the same sex sham. Gene expression fold change calculated using the 2^−ΔΔCt^ method, geometric mean of β-actin, Rpl3, and Rpl29 used as internal markers. Bar histograms show the mean ± SEM.

Differential expression analysis revealed an upregulation of Hmgb1 mRNA in the right CeA (*p* < 0.05, two-way ANOVA with Šidák’s post hoc tests), but not the left CeA of male SNL rats (*n* = 6) compared to sham control rats (*n* = 6), as shown by the normalized counts of the mapped reads for Hmgb1 in Figure 1B. There was a significant effect of the CeA hemisphere (F_1,20_ = 4.595; *p* < 0.05), as well as neuropathic pain (F_1,20_ = 10.14; *p* < 0.01) on Hmgb1 normalized counts. Based on this data, Hmgb1 was selected as a target for the validation of mRNA expression with qRT-PCR. Though bulk RNA sequencing was performed only on CeA tissues from male rats at the chronic stage of neuropathic pain, we elected to examine mRNA expression levels of Hmgb1 in the left and right CeA of male and female rats at both the 1-week acute and 4-week chronic stages of the SNL model (Figure 1C). In the right CeA at the chronic phase of neuropathic pain, there was a significant effect of SNL surgery on Hmgb1 mRNA expression (F_1,24_ = 27.09; *p* < 0.0001). We found significantly increased Hmgb1 mRNA expression in the right CeA of male (*n* = 6) and female (*n* = 6) rats compared to the same-sex sham (male, *n* = 9; female, *n* = 7) at the chronic SNL phase (male, *p* < 0.05; female, *p* < 0.001; two-way ANOVA with Šidák’s post hoc tests). No significant differences in Hmgb1 expression levels between SNL and sham rats were seen in the left CeA at the chronic stage, providing further evidence for pain-related changes and processing in the right rather than left amygdala. Hmgb1 mRNA levels did not significantly differ between SNL (male, *n* = 8; female, *n* = 8) and sham (male, *n* = 7; female, *n* = 9) rats at the acute stage of neuropathic pain for either sex in the right or the left CeA.

At the protein level, preliminary data (Figure S1) found that Hmgb1 was expressed in all neurons in the CeA (i.e., no NeuN+/Hmgb1− cells; see Figure S1A), including those that express corticotropin-releasing factors (CRFs), which are known to be projection neurons [13,19] that can modulate pain behaviors [50,51,52,53]. The data are consistent with neuronal original of Hmgb1 to modulate neuroimmune signaling in the amygdala in pain.

### 2.2. Effects of Hmgb1 siRNA Pre- and Post-Treatment in the CeA on Hmgb1 Expression

**Figure 2:**
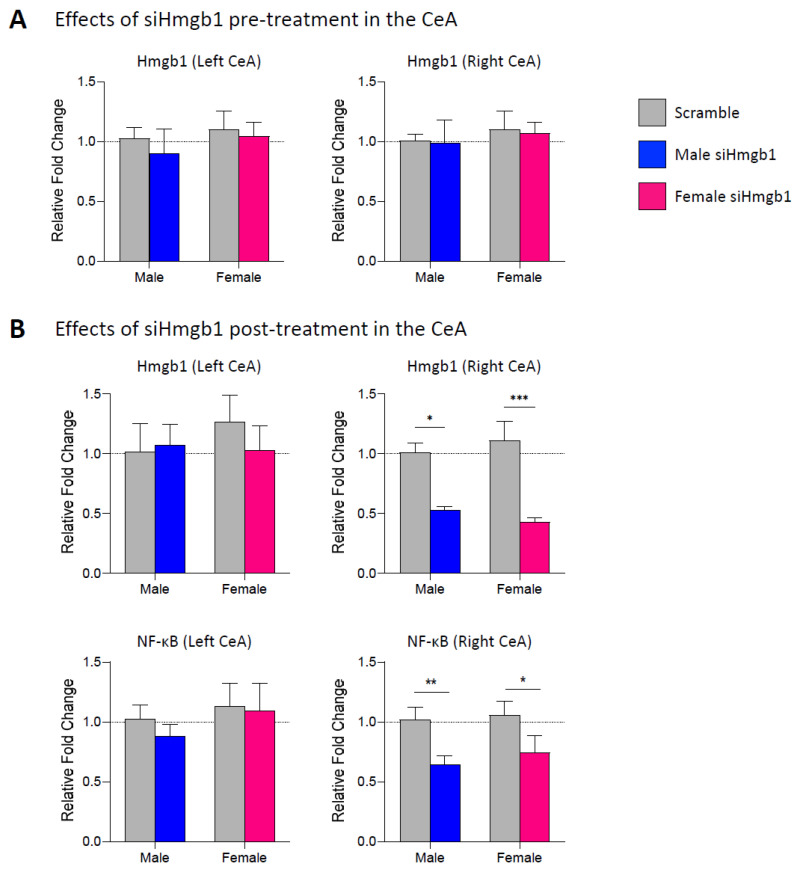
Effects of siHmgb1 pre- and post-treatment on mRNA expression levels in the CeA. (**A**) Hmgb1 siRNA AAV pooled viral vector (siHmgb1) or scrambled siRNA AAV control viral vector (scramble) was stereotaxically delivered into the right CeA of male and female rats as a 2-week pre-treatment (**A**) or 1-week post-treatment (**B**) to SNL surgery. At the chronic (4 week) phase of the SNL model, the brains were extracted and the left and right CeA were dissected out for mRNA validation. qRT-PCR analysis of mRNA expression levels for Hmgb1 was performed on left and right CeA tissues. (**A**) No significant differences were seen in Hmgb1 mRNA expression in either the left or the right CeA following siHmgb1 pre-treatment compared to scramble control for male (siHmgb1, *n* = 10; scramble, *n* = 8) and female (siHmgb1, *n* = 14; scramble, *n* = 14) SNL rats. (**B**) Hmgb1 mRNA expression was significantly downregulated in the right but not left CeA following siHmgb1 post-treatment compared to the scramble control in male (siHmgb1, *n* = 10; scramble, *n* = 6) and female (siHmgb1, *n* = 6; scramble, *n* = 10) SNL rats. mRNA expression of NF-κB, a downstream signaling molecule of Hmgb1, was also downregulated in the right but not left CeA following siHmgb1 post-treatment compared to the scramble control in male and female SNL rats. *, **, *** *p* < 0.05, 0.01, 0.001, two-way ANOVA with Šidák’s post hoc tests, compared to the same-sex scramble control. Gene expression fold change calculated using the 2^−ΔΔCt^ method, geometric mean of β-actin, Rpl3, and Rpl29 used as internal markers. Bar histograms show the mean ± SEM.

Next, we examined the mRNA expression levels of Hmgb1 in the left and right CeA following stereotaxic administration of siRNA for Hmgb1 (siHmgb1) or scrambled siRNA control viral vector (scramble) into the right CeA of male (siHmgb1, *n* = 10; scramble, *n* = 8) and female (siHmgb1, *n* = 14; scramble, *n* = 14) rats as a pre-treatment 2 weeks before SNL surgery (see Section 4.3 “Experimental protocol” and Figure 2A). The brains were extracted and the mRNA analysis performed at the chronic (4 week) stage of the SNL model (i.e., 6 weeks after siHmgb1 or scramble injection). We found no significant differences in Hmgb1 mRNA expression levels between siHmgb1-treated or scramble-treated SNL rats for either sex. Since our previous molecular studies indicated that Hmgb1 mRNA was only upregulated in the right CeA at the chronic stage of the SNL model (see Section 2.1 “Expression levels of Hmgb1 in the left and right CeA in neuropathic pain”), we then elected to allow Hmgb1 levels to become upregulated in our SNL model before targeting with siHmgb1. To do this, we stereotaxically administered siHmgb1 or scramble control into the right CeA of male (siHmgb1, *n* = 10; scramble, *n* = 6) and female (siHmgb1, *n* = 6; scramble, *n* = 10) rats as a 1-week post-treatment for SNL surgery, and collected tissues for mRNA analysis at the 4-week chronic stage of the SNL model (see Section 4.3 “Experimental protocol” and Figure 2B). We found that post-treatment with siHmgb1 significantly decreased Hmgb1 mRNA expression in the right but not left CeA of male and female SNL rats compared to the same-sex scramble control (male, *p* < 0.05; female, *p* < 0.001; two-way ANOVA with Šidák’s post hoc tests). We also examined mRNA expression levels in the CeA of NF-κB, a known downstream signaling molecule of Hmgb1 that can lead to the release of proinflammatory cytokines (see Figure 2B). Similar to Hmgb1, we found that NF-κB mRNA was significantly downregulated following siHmgb1 post-treatment in the right but not the left CeA in both sexes compared to the same-sex scramble control (male, *p* < 0.01; female, *p* < 0.05; two-way ANOVA with Šidák’s post hoc tests), supporting the efficacy of the siHmgb1 viral vector strategy in reducing the levels of Hmgb1 and downstream pro-inflammatory molecules in the right CeA in neuropathic pain.

**Figure 3:**
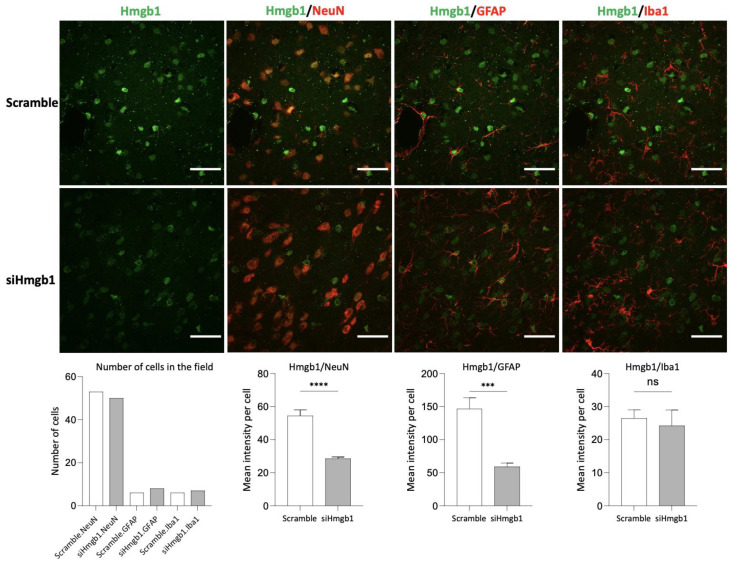
Cell type-specific effects of siHmgb1 post-treatment in the right CeA. Hmgb1 siRNA AAV pooled viral vector (siHmgb1) or scrambled siRNA AAV control viral vector (scramble) was stereotaxically delivered into the right CeA of male rats as a 1-week post-treatment to SNL surgery. For immunohistochemical studies, the brains were extracted from male rats at the chronic (4 weeks) phase of the SNL model (3 weeks after siHmgb1 or scramble injection). For each of the three cell types in the right CeA (neurons, GFAP+ astrocytes, and Iba1+ microglia), the Hmgb1 signal was evaluated by averaging the mean grey values (MGV) from each individual cell. Compared to rats injected with the scramble control viral vector, rats injected with siHmgb1 showed a reduction in Hmgb1 MGV per cell by 1.9 times in neurons and 2.5 times in astrocytes. There was no statistically significant difference in Hmgb1 signal for microglia. The field of analysis contained more neuronal cells than other cell types. ***, **** *p* < 0.001, 0.0001, ns, not significant, unpaired *t*-test. Error bars show the means *±* SEM. Scale bar, 40 μm.

At the protein level, the brains were extracted from male rats at the chronic (4 weeks) phase of the SNL model (i.e., 3 weeks after siHmgb1 or scramble injection). For each of the three cell types analyzed (neurons, GFAP+ astrocytes, and Iba1+ microglia), Hmgb1 signal was evaluated by averaging the mean grey values (MGV) from each individual cell. Compared to the rats injected with the scramble control, rats injected with siHmgb1 showed a reduction in Hmgb1 MGV per cell by 1.9 times in neurons and 2.5 times in astrocytes (neurons, *p* < 0.0001; astrocytes, *p* < 0.001; unpaired *t*-tests; see Figure 3). No statistically significant difference was seen in Hmgb1 MGV per cell in microglia following siHmgb1 versus scramble post-treatment. The decrease in Hmgb1 signal within the neuronal subgroup contributed more to the overall reduction in Hmgb1 signal between siHmgb1-treated and scramble-treated rats than any other cellular subgroup, as the field of analysis contained more neuronal cells than any other cell type (see Figure 3). Though the astrocytic population seemed to display a greater difference in Hmgb1 signal per cell than the neuronal population, this may be due to the large intensity emitting from a few select astrocytes in the field. 

### 2.3. Effects of Hmgb1 siRNA Pre-Treatment in the Right CeA on Chronic Neuropathic Pain Behaviors

**Figure 4:**
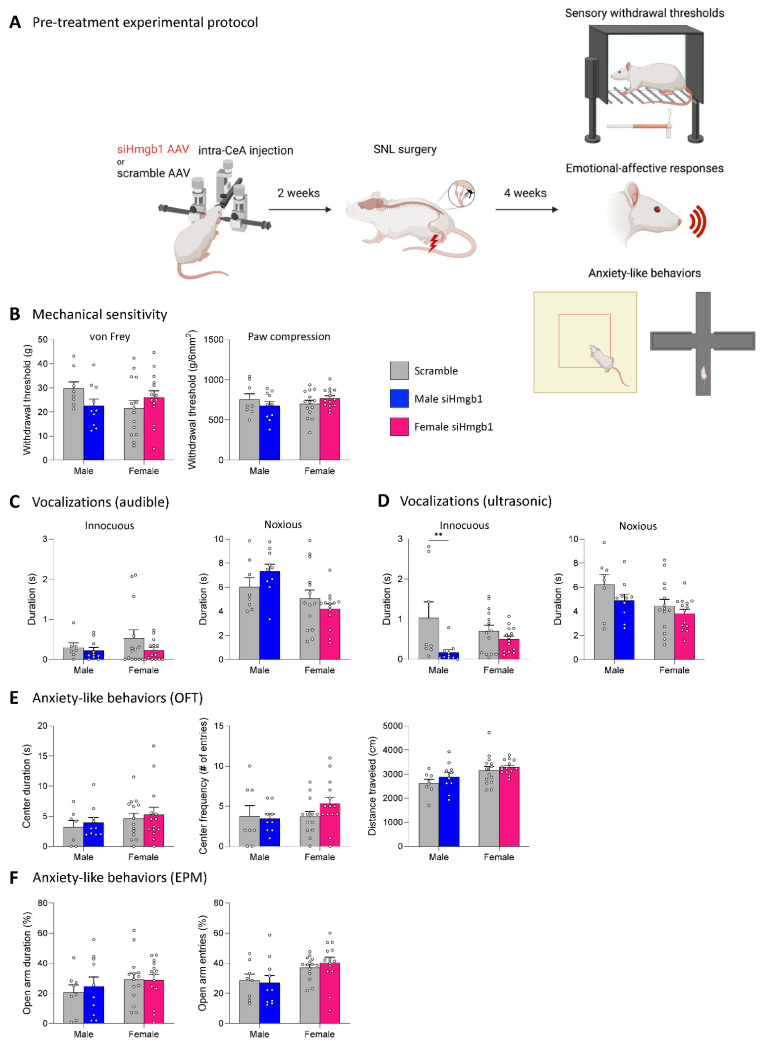
Effects of Hmgb1 siRNA pre-treatment in the right CeA on chronic neuropathic pain behaviors. (**A**) Hmgb1 siRNA AAV pooled viral vector (siHmgb1) or scrambled siRNA AAV control viral vector (scramble) was stereotaxically delivered into the right CeA of male (siHmgb1, *n* = 8; scramble, *n* = 10) and female (siHmgb1, *n* = 14; scramble, *n* = 14) rats as a pre-treatment 2 weeks before SNL surgery. Pain-related behavioral assays were performed 4 weeks after SNL surgery. (**B**) No significant differences were observed in siHmgb1 pre-treated male and female SNL rats in sensory withdrawal thresholds (measured by electronic von Frey and paw compression of the affected hind paw) compared to the scramble control. Emotional-affective responses were measured by the duration (s) of audible (**C**) or ultrasonic (**D**) vocalizations evoked by a brief (10 s) normally innocuous (100 g/6 mm^2^) or noxious (500 g/6 mm^2^) mechanical compression of the affected hind paw. Ultrasonic vocalization duration in response to innocuous stimulation was significantly decreased in siHmgb1 pre-treated males only (**D**); no significant effects were seen for males in audible vocalizations (**C**) or in females for audible (**C**) and ultrasonic (**D**) vocalizations. Anxiety-like behaviors measured by the center duration and number of center entries in the OFT, (**E**) and open arm duration and open arm entries in the EPM (**F**) were not significantly affected by siHmgb1 pre-treatment in male or female SNL rats compared to the scramble control. No significant differences were observed in the distance traveled within the OFT for male or female SNL rats following siHmgb1 pre-treatment compared to the scramble control (**E**). ** *p* < 0.01, two-way ANOVA with Šidák’s post hoc tests, compared to the same-sex scramble control. Bar histograms show the mean ± SEM. Experimental protocol figure created with BioRender.com, https://www.biorender.com/ (accessed on 8 May 2023).

Since Hmgb1 mRNA was only upregulated in the right CeA 4 weeks after SNL surgery (see Section 2.1 “Expression levels of Hmgb1 in the left and right CeA in neuropathic pain”), we selected the chronic stage of neuropathic pain and the right hemisphere as the focus of our behavioral studies. Our previous studies showed that males and females develop hypersensitivity, increased evoked vocalizations, and increased anxiety-like behaviors at the chronic stage of neuropathic pain (SNL model; [54]). To investigate the role of Hmgb1 in the right CeA in chronic neuropathic pain-related behavior, we stereotaxically administered siHmgb1 or scramble control into the right CeA of male (siHmgb1, *n* = 8; scramble, *n* = 10) and female (siHmgb1, *n* = 14; scramble, *n* = 14) rats as a pre-treatment 2 weeks before SNL surgery (see Section 4.3 “Experimental protocol” and Figure 4A). Behavioral assays were then performed 4 weeks after SNL surgery (i.e., 6 weeks after siHmgb1 or scramble injection) to assess the chronic phase of neuropathic pain.

We first examined mechanical sensitivity using two assays (see Section 4.6.1 “Mechanosensitivity”). With the electronic von Frey anesthesiometer, siHmgb1 pre-treatment had no significant effect on hind paw withdrawal thresholds in either male or female SNL rats compared to the scramble control (Figure 4B). Similarly, there were no significant differences in mechanical sensitivity in the paw compression test using calibrated forceps between siHmgb1-treated and scramble-treated SNL rats for either sex (see Figure 4B). For the statistical analyses of mechanical withdrawal thresholds in both the von Frey and the paw compression tests, a two-way ANOVA with Šidák’s post hoc tests was used (see Section 4.7 “Statistical analysis”).

Next, we evaluated emotional-affective responses by measuring the total duration of vocalizations in the audible (Figure 4C) and ultrasonic (Figure 4D) ranges in response to a normally innocuous or a noxious mechanical stimulus on the left hind paw (see Section 4.6.2 “Emotional-affective responses”). A lower stimulus intensity is sufficient to evoke vocalizations because of the increased sensitivity, reflected in a decreased threshold (see Section 4.6.2). siHmgb1 pre-treatment had no significant effect on the total duration of audible vocalizations evoked by normally innocuous or noxious stimuli in male or female SNL rats compared to the scramble control (Figure 4C). However, there was a significant effect of sex on audible vocalizations in response to noxious compression (F_1,42_ = 10.23; *p* = 0.0026), as female SNL rats vocalized less than male SNL rats following siHmgb1 pre-treatment. The total duration of ultrasonic vocalizations in response to a normally innocuous stimulus was significantly decreased for male (*p* < 0.01), but not female (*p* = 0.5951) SNL rats following siHmgb1 pre-treatment in comparison to the scramble control (Figure 4D). There was a significant effect of siHmgb1 treatment on ultrasonic vocalizations evoked by normally innocuous stimuli (F_1,42_ = 8.847; *p* = 0.0048). Conversely, the total duration of ultrasonic vocalizations evoked by a noxious stimulus was not significantly different between siHmgb1-treated and scramble-treated SNL rats in either sex (Figure 4D), though there was a significant effect of sex on ultrasonic vocalizations evoked by noxious stimulation (F_1,42_ = 6.593; *p* = 0.0139). For the statistical analyses of audible and ultrasonic vocalization durations, a two-way ANOVA with Šidák’s post hoc tests was used (see Section 4.7 “Statistical analysis”).

Finally, we assessed anxiety-like behaviors using the OFT and EPM (see Section 4.6.3 “Anxiety-like behaviors”). In the OFT, there were no significant differences in the time spent in the center zone or the number of entries into the center zone of the arena between SNL rats that received siHmgb1 and those that received the scramble control viral vector for either sex (Figure 4E). Importantly, no significant differences in locomotor activity in the OFT were observed between the siHmgb1-treated group and the scramble-treated group for SNL males (*p* = 0.4806) or for SNL females (*p* = 0.7291), indicating that both cohorts had similar levels of ambulatory activity (Figure 4E). Similarly, there was no significant effect of siHmgb1 pre-treatment on the time spent in the open arms or on the number of entries into the open arms in the EPM for either sex (Figure 4F); however, there was a significant effect of sex on open arm entries in the EPM (F_1,42_ = 8.448; *p* = 0.0058), with male SNL rats entering the open arms fewer times than their female SNL counterparts following siHmgb1 pre-treatment. For the statistical analyses of all locomotor activity and anxiety-like behavior in the OFT and EPM, a two-way ANOVA with Šidák’s post hoc tests was used (see Section 4.7 “Statistical analysis”).

### 2.4. Effects of Hmgb1 siRNA Post-Treatment in the Right CeA on Chronic Neuropathic Pain Behaviors

**Figure 5:**
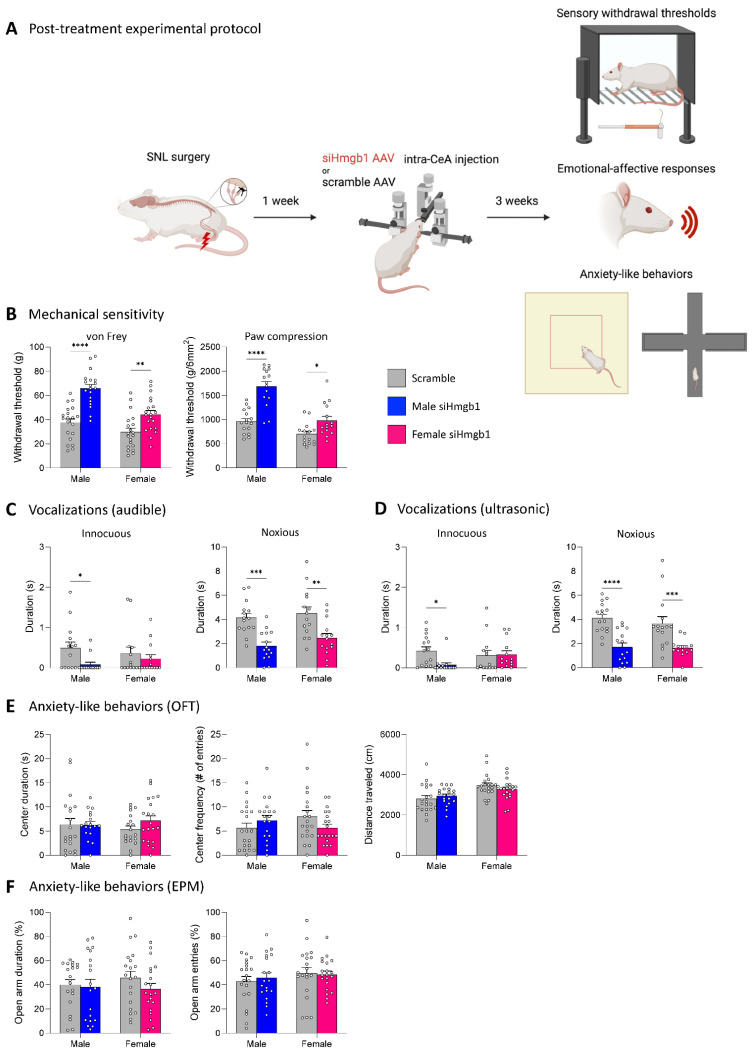
Effects of Hmgb1 siRNA post-treatment in the right CeA on chronic neuropathic pain behaviors. (**A**) Hmgb1 siRNA AAV pooled viral vector (siHmgb1) or scrambled siRNA AAV control viral vector (scramble) was stereotaxically delivered into the right CeA of male (siHmgb1, *n* = 19; scramble, *n* = 20) and female (siHmgb1, *n* = 20; scramble, *n* = 20) rats as a post-treatment 1 week after SNL surgery. Pain-related behavioral assays were performed 4 weeks after SNL surgery. (**B**) Sensory withdrawal thresholds (measured by electronic von Frey and paw compression of the affected hind paw) were increased by siHmgb1 post-treatment in male and female SNL rats compared to the scramble control. Emotional-affective responses were measured by the duration (s) of audible (**C**) or ultrasonic (**D**) vocalizations evoked by a brief (10 s) normally innocuous (100 g/6 mm^2^) or noxious (500 g/6 mm^2^) mechanical compression of the affected hind paw. Audible and ultrasonic vocalization duration in response to normally innocuous stimulation was significantly decreased in siHmgb1 post-treatment males only; audible and ultrasonic vocalization duration evoked by noxious stimulation was significantly decreased in both males and females following siHmgb1 post-treatment when compared to the scramble control. Anxiety-like behaviors measured by center duration and number of center entries in the OFT (**E**) and open arm duration and open arm entries in the EPM (**F**) were not significantly affected by siHmgb1 post-treatment in male or female SNL rats compared to the scramble control. No significant differences were observed in the distance traveled in the OFT for male or female SNL rats following siHmgb1 post-treatment when compared to the scramble control (**E**). *, **, ***, **** *p* < 0.05, 0.01, 0.001, 0.0001, two-way ANOVA with Šidák’s post hoc tests, compared to the same-sex scramble control. Bar histograms show the mean ± SEM. Experimental protocol figure created with BioRender.com, https://www.biorender.com/ (accessed on 8 May 2023).

Since siHmgb1 pre-treatment had no molecular or behavioral effects, except on ultrasonic vocalizations in males, we elected to test the effects of Hmgb1 knockdown after SNL induction (post-treatment paradigm). In support of this strategy, Hmgb1 has been shown to become more strongly expressed in the cerebrospinal fluid [55] and spinal dorsal horn [55,56] throughout neuropathic pain development. To investigate the role of Hmgb1 in the CeA on chronic neuropathic pain-related behavior, we stereotaxically administered siHmgb1 or the scramble control viral vector into the right CeA of male (siRNA, *n* = 19; scramble, *n* = 20) and female (siRNA, *n* = 20; scramble, *n* = 20) rats as a post-treatment 1 week after SNL surgery (see Section 4.3 “Experimental protocol” and Figure 5A). Behavioral assays were then performed 3 weeks after siHmgb1 or scramble injection (i.e., 4 weeks after SNL induction) to assess the chronic phase of neuropathic pain.

We first evaluated mechanical sensitivity (see Section 4.6.1 “Mechanosensitivity”). In the von Frey test, siHmgb1 injection into the right CeA significantly increased the hind paw withdrawal thresholds in both male (*p* < 0.0001) and female (*p* < 0.01) SNL rats compared to the scramble control (Figure 5B). There was a significant effect of siHmgb1 post-treatment (F_1,75_ = 44.54; *p* < 0.0001) and a significant effect of sex (F_1,75_ = 20.73; *p* < 0.0001), as well as a significant interaction between the two factors (F_1,75_ = 4.572; *p* = 0.0358) on hind paw withdrawal thresholds. Similarly, siHmgb1 administration into the right CeA significantly decreased mechanical sensitivity in the paw compression test using calibrated forceps in both male (*p* < 0.0001) and female (*p* < 0.05) SNL rats compared to the scramble control (Figure 5B). There was a significant effect of siHmgb1 post-treatment (F_1,57_ = 40.54; *p* < 0.0001) and a significant effect of sex (F_1,57_ = 37.22; *p* < 0.0001), as well as a significant interaction between the two factors (F_1,57_ = 7.955; *p* = 0.0066) on withdrawal thresholds. For the statistical analyses of mechanical withdrawal thresholds in both the von Frey and paw compression tests, a two-way ANOVA with Šidák’s post hoc tests was used (see Section 4.7 “Statistical analysis”).

Next, we examined emotional-affective responses by measuring the total duration of audible (Figure 5C) and ultrasonic (Figure 5D) vocalizations in response to either a normally innocuous or noxious stimulus on the left hind paw (see Section 4.6.2 “Emotional-affective responses”). siHmgb1 administration significantly decreased the total duration of audible (*p* < 0.05, see Figure 5C) and ultrasonic (*p* < 0.05, Figure 5D) vocalizations in response to innocuous paw compression in male but not female (audible, *p* = 0.7237; ultrasonic, *p* = 0.9870) SNL rats compared to the scramble control. There was a significant effect of siHmgb1 post-treatment (F_1,53_ = 4.814; *p* = 0.0326) on audible vocalizations evoked by innocuous stimulation. siHmgb1 decreased the total duration of audible and ultrasonic vocalizations evoked by a noxious stimulus in SNL rats of both sexes compared to the scramble control (audible: male, *p* < 0.001; female, *p* < 0.01, Figure 5C; ultrasonic: male, *p* < 0.0001; female, *p* < 0.001, Figure 5D). There was a significant effect of siHmgb1 post-treatment on audible (F_1,55_ = 29.89; *p* < 0.0001) and ultrasonic (F_1,56_ = 34.61; *p* < 0.0001) vocalizations in response to noxious hind paw compression. For the statistical analyses of audible and ultrasonic vocalization durations in response to innocuous and noxious stimulation of the left hind paw, a two-way ANOVA with Šidák’s post hoc tests was used (see Section 4.7 “Statistical analysis”).

Finally, we assessed anxiety-like behaviors in the OFT and EPM (see Section 4.6.3 “Anxiety-like behaviors”). In the OFT, there were no significant differences in the time spent or in the number of entries in the arena center zone between siHmgb1-treated and scramble-treated SNL rats for either sex (Figure 5E). It is important to note that there were also no significant differences in the distance traveled within the OFT arena between the siRNA and scramble cohorts for SNL males (*p* = 0.7214) or SNL females (*p* = 0.4680), signifying that all groups had similar levels of ambulatory activity within the assay (Figure 5E). Similarly, siHmgb1 post-treatment did not have a significant effect on the time spent in the open arms or on the number of entries into the open arms of the EPM for either sex (Figure 5F). For the statistical analyses of all anxiety-like behavior and locomotor activity in the OFT and EPM, a two-way ANOVA with Šidák’s post hoc tests was used (see Section 4.7 “Statistical analysis”).

## 3. Discussion

This study explored the role of Hmgb1 in the CeA on sensory, emotional-affective, and anxiety-like pain-related behaviors in male and female rats in a neuropathic pain model. It has previously been demonstrated that Hmgb1 is likely implicated in the pathogenesis of neuropathic pain in the periphery and spinal cord [36,37,38]. However, it is unclear if these findings extend into brain regions that are implicated in pain processing and pain modulation, such as the amygdala. The key novelties of this study are the characterizations of Hmgb1 expression levels in the CeA at the acute and chronic stages of neuropathic pain in both sexes, and the right-hemispheric lateralization of Hmgb1 increase at the chronic neuropathic pain stage; the differential behavioral modulation of Hmgb1 knockdown in the right CeA preceding and following neuropathic pain induction; and the proposed hypothesis of neuronally driven glial activation through proinflammatory mediators such as Hmgb1 within the CeA in a chronic neuropathic pain state.

Both preclinical [10,11,12,13,15,19,57] and clinical [58,59,60,61,62,63,64,65,66,67,68] studies have highlighted the role of the amygdala in pain processing and pain modulation. In particular, the lateral and capsular regions of the CeA have been regarded as the “nociceptive amygdala” in reference to the abundance of neurons in these regions that encode nociceptive information and modulate pain-related behaviors [11,13,18]. Changes in neuronal activity within these regions have been noted in the following pain models: inflammatory pain [69,70,71,72,73], muscular pain [74], arthritic pain [45,75,76,77,78,79,80,81,82,83,84,85], visceral pain [86,87,88,89], functional pain [50], and neuropathic pain [15,27,90,91,92,93,94,95]. Conversely, the inhibition of amygdala activity has been demonstrated to reduce pain-related behaviors in inflammatory [73,95,96,97,98,99], arthritic [45,77,82,100,101,102,103,104,105,106,107,108,109], visceral [87], widespread nociplastic [110], chemotherapy-associated [111], and neuropathic pain models [15,27,52,90,95,112,113,114,115,116]. Less clear is the role of amygdala neuroplasticity and underlying mechanisms in the transition from acute to chronic pain. Therefore, a thorough investigation of pain modulatory mechanisms, particularly within the CeA, is warranted.

Accumulating evidence suggests pain-related hemispheric laterization of amygdala function. Whereas the right CeA has generally been shown to play a more pro-nociceptive role [14,44,45], pain-related functioning of the left CeA is not as clear. In the kaolin/carrageenan arthritis pain model, neurons in the right but not left latero-capsular division of the CeA developed increased background activity and evoked responses, irrespective of the side of arthritis induction [45], suggesting a right CeA predominance in arthritic pain. Similarly, pain-like behaviors from left-sided neuropathic pain models have been associated with increases in excitatory synaptic transmission from parabrachial afferents to the right CeA [91,92]. There has been some evidence to suggest that time-dependent differences in neuronal activation exist between the two hemispheres in the development of the chronic state after nerve injury. One study found that increases in spontaneous and evoked neuronal activity were higher in the left CeA when compared to the right 2–6 days after SNL induction, whereas this activity became dominant in the right CeA 14 days after surgery [92], possibly indicating different functional states within the two regions in the transition from acute to chronic pain. Overwhelming evidence for a right CeA-predominant role in inflammatory pain modulation has likewise been demonstrated, as a blockade of the formalin-induced extracellular signal-regulated kinase (ERK) cascade in the right but not left CeA decreased mechanosensitivity [44]. In inflammatory pain models, synaptic transmission from the parabrachial nucleus was potentiated only in the right CeA regardless of the side of inflammation induction [70,72]. Calcitonin gene-related peptide (CGRP), a neuropeptide that is highly involved in pain processing and considered an important molecular marker of the “nociceptive amygdala” [71,117,118,119,120], has differential consequences in the left and right CeA. An infusion of CGRP increased pain-like behaviors (mechanosensitivity and emotional-affective responses [121] and bladder pain-like visceromotor responses [88]) when injected into the right CeA of naïve rats, whereas delivery into the left CeA of naïve rats increased mechanical withdrawal thresholds [122] and decreased visceral pain behaviors [88]. As CGRP release from sensory neurons likely influences many downstream inflammatory molecules, including Hmgb1 [123], it is reasonable to infer that these neuronally driven hemispheric differences in pain modulation can impact the expression and function of neuroinflammatory mediators in the right and left CeA differently.

Hmgb1 expression is widely distributed in the peripheral and central nervous systems. While present in neurons and Schwann cells of the periphery [124], Hmgb1 has also been found in neurons, astrocytes, and microglia of the central nervous system [40,125,126,127]. Within the rodent brain, Hmgb1 levels have been reported in regions such as the hippocampus [128,129,130,131,132,133], prefrontal cortex [41,130,132,134,135], anterior cingulate cortex (ACC) [136], hypothalamus [137,138,139], and amygdala [41,140,141]. Numerous studies have explored Hmgb1 mRNA and protein expression levels throughout pain development within the periphery and spinal cord. In the dorsal root ganglion (DRG), increased Hmgb1 expression levels were reported in primary afferent neurons and satellite glial cells 1 day after SNL induction [142], in primary afferent neurons 14 days after tibial nerve injury (TNI) [42], and in infiltrating macrophages and proliferating Schwann cells 14 days after partial sciatic nerve ligation (PSNL) [143]. Hmgb1 expression levels have been reportedly upregulated in spinal cord dorsal horn neurons at 21 (but not at 3) days post-PSNL [144], though another study found that Hmgb1 protein was markedly increased in the same region both 3 and 7 days after chronic constriction injury (CCI) induction [145]. Late-stage increases in spinal Hmgb1 expression levels have also been reported in the CCI model of neuropathic pain [146]. Despite this abundant evidence to support Hmgb1′s role in pain modulation in the DRG and spinal cord, its specific involvement in pain processing within the brain has not been extensively researched. Hmgb1 was found to be upregulated predominately in medial prefrontal cortex (mPFC) neurons 9 days after partial infraorbital nerve transection (p-IONX) [41]; the same study found sustained Hmgb1 protein upregulation in the basolateral nucleus of the amygdala (BLA; hemisphere unspecified) 9–30 days after neuropathic pain induction, though the source of Hmgb1 was not explored within this region. In inflammatory pain induced by complete Freund’s adjuvant (CFA), Hmgb1 protein was upregulated in the ACC 7 days after pain induction [136]. 

To our knowledge, the current study is the first to report neuropathic pain stage-specific mRNA expression levels of Hmgb1 in the right and left CeA. Our mRNA expression data support a role for Hmgb1 at the chronic phase of neuropathic pain in both male and female rats. Previously mentioned studies suggest that Hmgb1 may act as a critical regulator in pain maintenance at other levels of the neuraxis [42,143,144,146]. Our results indicate that within the amygdala, knockdown of Hmgb1 with siRNA not only decreased Hmgb1 expression, but also NF-κB expression, which may lead to a decreased release of pro-inflammatory cytokines that can drive pain behaviors; therefore, Hmgb1 in the amygdala may also play a similar role in pain modulation, particularly at the chronic stage. Importantly, this modulatory mechanism appears to play a predominant role in the right rather than the left CeA, providing further support for right-hemispheric lateralization of pain processing within the amygdala. Additional investigation is needed to confirm temporal differences in Hmgb1 expression at the protein level in both hemispheres of male and female rats. 

We report that Hmgb1 knockdown in the right CeA inhibits evoked sensory and emotional responses, but not anxiety-like behaviors, in support of the literature that suggests that Hmgb1 is a pathogenic mediator of pain-related behaviors [36,37,38]. Delivery of Hmgb1 into the periphery [142,147,148,149] and spinal cord [127,150] has been shown to induce mechanical hypersensitivity in rodents of both sexes. In contrast, blocking the actions of Hmgb1 has been explored at all levels of the neuraxis using a variety of inhibitory techniques. Neutralization with an anti-Hmgb1 antibody has been shown to inhibit mechanical allodynia in neuropathic pain models when injected systemically [41,144,151,152] or peripherally [142], and in inflammatory pain models when injected peripherally [149] or intrathecally [127]; it has also been utilized to reduce mechanical sensitivity associated with models of type 2 diabetes [153], pancreatitis [154], bone cancer [155], and bladder pain [156]. Other approaches to decrease Hmgb1 action to reduce pain-like behaviors have included inhibition with glycyrrhizin, a natural anti-inflammatory triterpene that binds to Hmgb1 [42,136,157,158]; recombinant human soluble thrombomodulin, which sequesters Hmgb1 and promotes its degradation [149,154,156,159]; recombinant Hmgb1 box A peptide, which antagonizes Hmgb1 through an unknown mechanism [127]; and more recently, through various microRNAs (miRNAs) or long non-coding RNAs (lncRNAs) that directly target Hmgb1 to negatively regulate its expression [160,161,162,163,164]. However, experimental protocols for the delivery of these Hmgb1 inhibitors (pre- versus post-pain induction, single versus multiple doses, etc.) vary widely between studies, rendering it difficult to draw firm conclusions regarding the optimal time point for pain relief. Of note, though the current study found no beneficial effects of administering siHmgb1 as a pre-treatment to neuropathic pain induction (aside from a decrease in ultrasonic vocalizations following low intensity stimulation in neuropathic males), others have reported decreases in mechanical hypersensitivity following pre-treatment with an Hmgb1 inhibitor [149,156,162,163], though in some cases this was followed by repeated injections after pain induction [142]. The discrepancy in behavioral effects following siHmgb1 pre- and post-treatment in the current study may be attributed to the delayed upregulation of Hmgb1 in neuropathic pain (i.e., increased expression must occur before becoming a viable therapeutic target), or potentially to the difference in duration between the siHmgb1 injection and behavioral assays (i.e., a single injection of siHmgb1 may not have had sufficient long-lasting impacts on pain behavior in the pre-treatment group).

Interestingly, one group found that while inhibition with an anti-Hmgb1 antibody bilaterally infused into the mPFC for 9 days following neuropathic pain induction had no effect on hind paw mechanical thresholds, anxiety-like behaviors in the EPM were significantly alleviated [41]. The same study reported no effect on anxiety-like behavior when the anti-Hmgb1 antibody was injected into the bilateral BLA, however, suggesting that Hmgb1 upregulation in the mPFC as opposed to the BLA is required for anxiety onset in neuropathic pain [41]. This is in agreement with the results presented in this study that show that Hmgb1 knockdown in the CeA had no effect on anxiety-like behaviors of neuropathic rats. Though it has been reported that an intracerebroventricular delivery of recombinant Hmgb1 can induce anxiodepressive-like behaviors in a neuropathic pain state [165], our results suggest that this may not be attributed to actions within the amygdala. Importantly, to our knowledge this study is the first to report that Hmgb1 inhibition in the amygdala may also reduce the emotional-affective dimension of pain, as well as mitigating the sensory component. Our behavioral results extend the existing evidence for a pro-nociceptive role of Hmgb1 in chronic pain conditions to the amygdala, specifically at delayed stages of pain development. The inhibition of intra-amygdala Hmgb1 at this time point is advantageous in that it may address multiple facets of the multidimensional nature of pain, rendering this therapeutic approach an appealing strategy for chronic neuropathic pain relief.

Hmgb1 has been well established as a critical and prominent mediator of inflammation and immunity [33,46]. Hmgb1 may be passively and instantaneously released from damaged or dying cells through mechanisms associated with increased cell permeability [166,167,168]. However, under profound cellular stress its active secretion is initiated by membrane receptor interactions and slower intracellular signaling transduction cascades [169,170,171]. Several studies have specifically highlighted Hmgb1 release from neurons [172,173,174,175], possibly through mechanisms involving a subcellular redistribution from the nucleus to the cytoplasm [42,152]. This may then act on glial receptors such as RAGE, TLR2, and TLR4 to increase the transcription of NF-κB and release of proinflammatory cytokines, accelerate neuroinflammation, and further Hmgb1 release [40]. In turn, Hmgb1 has been repeatedly shown to induce neuronal hyperexcitability, specifically in the DRG and mPFC [41,42,43]. As persistent neuronal hyperactivity has been associated with central sensitization in neuropathic pain [176,177,178,179], it is possible that Hmgb1 release within the CeA may act as a critical mechanistic factor in neuropathic pain development and/or maintenance (see graphical abstract). Preliminary evidence shows that Hmgb1 is expressed predominantly on neurons in the CeA (see Figure S1A), and that this includes co-localization on CRF neurons (see Figure S1B). CRF neurons within the amygdala act as both long-range output projections, as well as local influencers on CeA and BLA circuitry [13,180], in addition to serving as critical modulators of pain-related neuroplasticity and behavior [11,50,51,52,53,81,103,181]. Therefore, Hmgb1 release from these neurons may have important functional implications in neuropathic pain pathogenesis. Further exploration is needed to determine precise cell type-specific and temporal mechanisms and downstream targets of Hmgb1 signaling within the CeA in a neuropathic pain state.

It is important to note that while no major sex differences in Hmgb1-related pain-like behaviors were observed in the amygdala in the present study, a few sex-dependent roles for Hmgb1 have been noted previously. In the periphery, the blockade of Hmgb1 with an Hmgb1-neutralizing antibody reduced mechanical hypersensitivity in male but not female mice in a collagen antibody-induced arthritis (CAIA) model, despite intra-articular injection of disulfide Hmgb1 evoking mechanical hyperalgesia in naïve mice of both sexes [147]. This corresponded to temporal differences in the mRNA levels of several proinflammatory factors, with most peaking in females 2 h after Hmgb1 injection and returning to baseline by the 4 h timepoint; in contrast, males showed a more pronounced increase at the 4 h timepoint that was significantly higher than females 6 h after injection [147]. The blockade of resident macrophages by the inhibitor minocycline protected male but not female mice from developing Hmgb1-induced hypersensitivity [147]. Similar results were observed in the spinal cord, where intrathecal administration of minocycline co-administered with Hmgb1 prevented pain behavior only in male mice, and higher cytokine responses were observed in microglia derived from males compared to females following Hmgb1 administration [182]. Together, these data suggest that Hmgb1 and subsequent glial activation may play a more critical role for males than females in peripheral and spinal pain mechanisms. Our previous study suggested that mechanisms other than CGRP-related neuronal signaling may be more relevant for amygdala pain processing in males compared to females [112]; this could potentially include neuroimmune signaling interactions related to Hmgb1, as the majority of the pain-related behaviors presented in the current study were more strongly impacted in males compared to females following Hmgb1 inhibition. However, underlying pain processing mechanisms for females are complex and not well-characterized, particularly within the brain. This highlights the urgent need for further exploration into sex-specific neuroimmune modulation at all levels of the neuraxis [183].

## 4. Materials and Methods

### 4.1. Animals

Adult male and female Sprague-Dawley rats (150–300 g, 8 weeks of age at the start of experiments; 250–400 g, 12 weeks of age at time of behavioral testing) were group-housed (*n* = 3 per cage) in a temperature-controlled room under a 12 h day/night cycle with ad libitum access to food and water. On each experimental day, the rats were transferred from the animal facility and allowed to acclimate to the laboratory for at least 1 h prior to testing. All experimental procedures were approved by the Institutional Animal Care and Use Committee (IACUC, protocol #21026) of Texas Tech University Health Sciences Center (TTUHSC) and conformed to the guidelines of the International Association for the Study of Pain (IASP) and the National Institutes of Health (NIH). Male and female rats were randomly assigned to the different experimental groups. All behavioral studies were performed with the experimenter blinded to the treatment conditions.

### 4.2. Neuropathic Pain Model

The well-established spinal nerve ligation (SNL) rat model of neuropathic pain [184] was utilized to induce a stable and long-lasting peripheral neuropathy. Rats were anesthetized with isoflurane (2–3%; precision vaporizer, Harvard Apparatus, Holliston, MA, USA) and underwent surgery in which the left L5 spinal nerve was exposed and tightly ligated using 6–0 sterile silk. A sham-operated control group underwent a similar surgical procedure where the L5 spinal nerve was exposed but not ligated. Topical antibiotic (Bacitracin) was applied daily for 5 days after all surgical procedures to prevent infection.

### 4.3. Experimental Protocol

The gene expression levels in the left and right CeA of male rats at the chronic phase (4 weeks post-SNL or -sham surgery) of neuropathic pain (see Section 4.2 “Neuropathic pain model”) were measured using bulk RNA sequencing and quantified during differential gene expression analysis (see Section 4.4 “Gene expression using bulk RNA sequencing”). Hmgb1 was selected as a target gene of interest and the mRNA expression levels of Hmgb1 were measured (see Section 4.5 “qRT-PCR and immunohistochemistry”) in the left and right CeA of male and female rats at the acute (1 week post-SNL or -sham surgery) and chronic (4 weeks post-SNL or -sham surgery) phases of neuropathic pain. The molecular and behavioral effects of an Hmgb1 AAV siRNA viral vector compared to the scrambled control AAV viral vector were tested in male and female rats 4 weeks after SNL surgery following a single stereotaxic injection into the right CeA either at 2 weeks before or 1 week after SNL surgery (see Section 4.5 “qRT-PCR and immunohistochemistry” and Section 4.6 “Behaviors”). For stereotaxic injection, male and female rats were anesthetized with isoflurane (2–3%; precision vaporizer, Harvard Apparatus, Holliston, MA, USA) and a small unilateral craniotomy was performed as described previously [50,90,112]. A stereotaxic apparatus (David Kopf Instruments, Tujunga, CA, USA) was used to inject 1 µL of either Hmgb1 AAV siRNA viral vector of 4 pooled oligomers (serotype 5) (originally purchased as cat. #234960960215, now cat. #23496164, customized to serotype; Applied Biological Materials, Richmond, BC, Canada; target sequences: 70 CGGGAGGAGCACAAGAAGAAGCACCCGGA, 196 GCTGACAAGGCTCGTTATGAAAGAGAAAT, 365 TTGGTGATGTTGCAAAGAAACTAGGAGAG, 442 GCTGCCAAGCTGAAGGAGAAGTATGAGAA) or the scrambled AAV siRNA control viral vector (serotype 5) (cat. #iAAV01505; Applied Biological Materials) over a 10 min period, using a 5 μL Hamilton syringe for the right CeA, using the following coordinates [185]: 2.5 mm caudal to the bregma, 4.3 mm lateral to the midline, and 7.6 mm deep. Topical antibiotic (Bacitracin) was applied daily for 5 days following the craniotomy to prevent infection.

### 4.4. Gene Expression Using Bulk RNA Sequencing

At the chronic phase (4 weeks post-SNL or -sham surgery) of neuropathic pain (see Section 4.2 “Neuropathic pain model”), male rats (SNL, *n* = 6; sham, *n* = 6) were euthanized via decapitation. The brains were rapidly extracted and oxygenated in ice-cold artificial cerebrospinal fluid (ACSF) that contained the following (in mM): 125.0 NaCl, 2.6 KCl, 2.5 NaH_2_PO_4_, 1.3 CaCl_2_, 0.9 MgCl_2_, 21.0 NaHCO_3_, and 3.5 glucose. Coronal brain slices (1000 μM) containing the left and right CeA were prepared using a Vibratome (VT1200S, Leica Biosystems, Nussloch, Germany) as described previously [51,112,186]. The left and right CeA were dissected from freshly harvested slices for bulk RNA sequencing analysis (see diagram of a coronal brain slice containing the dissected amygdala regions in Figure 1A). Total RNA was isolated using the MagMAXTM-96 Kit (Life Technologies, Carlsbad, CA, USA) and checked for quality control (all RIN values were >8.4). RNA library preparation and sequencing were performed at the University of Texas at Austin Genomic Facility (https://wikis.utexas.edu/display/gsaf [accessed on 15 March 2019]). Illumina TagSeq of poly-A enriched total RNA sequencing was performed (single end, 100 bp). Individual sample libraries were mapped to *Rattus norvegicus* (Rnor_6.0; https://useast.ensembl.org/Rattus_norvegicus/Info/Index [accessed on 8 June 2022]) reference genome using STAR Aligner [187]. Aligned sequencing reads were quantified using StringTie (v2.2.1). Quantified expression data were analyzed for differential expression between SNL and sham animals for both the left and right CeA using the R Bioconductor package DESeq2 (v1.36.0) [188] within RStudio (v4.2.0), producing fold change, *p*-values, and estimated false discovery rate (FDR). Candidate differentially expressed genes (DEGs) were identified using a 5% FDR threshold and a convergent validity approach that combines nominal statistical significance and the biological significance of bioinformatics analysis to control for Type 1 and Type 2 error rates, as described previously [189]. Identified DEGs were screened for mechanistic candidates that may contribute to cellular changes in nociceptive processing. There was a specific focus on DEGs that are known to be involved in neuroimmune interactions in other clinical disorders, as current knowledge of neuroimmune signaling mechanisms in pain conditions largely stems from research on peripheral and spinal nociceptive processing. Within these criteria, Hmgb1 was selected as a potential factor of interest for investigation as a mechanism and therapeutic target for chronic neuropathic pain. The RNA sequencing results presented in this study are preliminary to allow us to highlight the role of Hmgb1 in neuropathic pain, and a more comprehensive analysis of this dataset will be presented in an upcoming manuscript.

### 4.5. qRT-PCR and Immunohistochemistry 

At either the acute phase (1 week post-SNL or -sham surgery) or chronic phase (4 weeks post-SNL or -sham surgery) of neuropathic pain (see Section 4.2 “Neuropathic pain model”), male and female rats were euthanized via decapitation. The brains were rapidly extracted and oxygenated in ice-cold sucrose-based physiological solution that contained the following (in mM): 87 NaCl, 75 sucrose, 25 glucose, 5 KCl, 21 MgCl_2_, 0.5 CaCl_2_, and 1.25 NaH_2_PO_4_. Coronal brain slices (1000 μm) containing the left and right CeA were prepared using a Vibratome (VT1200S, Leica Biosystems) as described previously [51,112,186]. The left and right CeA were dissected from freshly harvested slices for separate mRNA expression analysis. RNA was extracted using the MagMAXTM-96 Kit (Life Technologies) and quantified on a NanoDrop 8000 spectrophotometer (Thermo Fisher Scientific, Rockford, IL, USA). Total RNA was reverse transcribed using the High-Capacity cDNA Reverse Transcription Kit with RNase Inhibitor (Thermo Fisher Scientific) and Taqman Fast Advanced Master Mix (Thermo Fisher Scientific) was used to perform quantitative reverse transcription polymerase chain reactions (qRT-PCR). Applied Biosystems Taqman Gene Expression Assays included Hmgb1 (*Hmgb1*; Rn02377062_g1), β-actin (*Actb*; Rn00667869_m1), ribosomal protein L3 (*Rpl3*; Rn01505100_g1), and ribosomal protein L29 (*Rpl29*; Rn00820801_g1). Reactions containing 5 ng of cDNA were performed in duplicate using the CFX384 Real-Time System (BioRad, Hercules, CA, USA). Relative expression was determined using the 2^−ΔΔCt^ method with samples normalized to the geometric mean of β-actin, Rpl3, and Rpl29. This normalization strategy utilized three genes that have previously been shown to have the most stable expression in a rat neuropathic pain model [190] and that have been reliable reference markers to analyze gene expression within the CeA tissue of neuropathic rats in our prior study [112].

For immunohistochemical studies, male rats were deeply anesthetized (4% isoflurane; precision vaporizer, Harvard Apparatus) and transcardially perfused with 0.1 M phosphate-buffered saline (PBS), followed by 4% paraformaldehyde (PFA) in PBS. Brains were extracted and fixed at 4 °C overnight in 4% PFA, then cryoprotected in a 30% sucrose solution for 48 h and embedded in optimal cutting temperature (OCT) compound. Coronal (40 μm) sections were prepared using a cryostat (HM525NX, Epredia, Kalamazoo, MI, USA). Sections were permeabilized in 0.3% Triton in PBS (PBST) for 10 min, blocked in 5% Normal Goat Serum (NGS) in PBS for 1 h, and incubated with the primary antibodies (α-Hmgb1, Mouse, R&D Systems, Minneapolis, MN, USA, #MAB1690, 1:100 dilution; α-NeuN (neuron-specific nuclear protein), Guinea Pig, Millipore, Burlington, MA, USA, ABN90, 1:1000 dilution; α-GFAP, Chicken, Novus, NBP1-05198, 1:1000 dilution; α-Iba1, Rabbit, Wako, 019-19741, 1:1000 dilution) with 5% NGS in PBS overnight. The next day, the sections were washed 3 × 5 min in PBST and then incubated in secondary antibodies (Goat-anti-Mouse, Alexa 405, A48255; Goat-anti-Guinea Pig, Alexa 647, A21450; Goat-anti-Chicken, Alexa 488, AA11039; Goat-anti-Rabbit, Alexa 568, A11011; Thermo Fisher) for 2 h at room temperature. After washing 3 × 5 min in PBS, sections were mounted in ProLong antifade media (P36961, Thermo Fisher). For the quantification of Hmgb1+ cells, sections were imaged using a confocal microscope (Olympus FV3000, Tokyo, Japan) with 60× oil immersion objective in four channels: Hmgb1, NeuN, GFAP, and Iba1 in the right CeA region. Image analysis was performed with ImageJ software (v1.53f51, NIH, Bethesda, MD, USA). For each cell type, the mean gray value in individual cells was measured.

### 4.6. Behaviors

All behavioral assays were performed in male and female rats 4 weeks after SNL surgery in both the Hmgb1 siRNA pre-treatment and post-treatment groups (see Section 4.3 “Experimental protocol”).

#### 4.6.1. Mechanosensitivity

Mechanical withdrawal thresholds were measured using a plantar electronic von Frey anesthesiometer (IITC Life Sciences, Woodland Hills, CA, USA) with the tip applied perpendicularly with increasing force to the base of the 3rd toe on the left hind paw (site of injury) as described previously [52,112]. Force was applied until a flexion reflex was provoked, and this was automatically recorded as the paw withdrawal threshold (in grams). Three threshold measurements were recorded and averaged to determine the final threshold value. Thresholds for sham-operated rats at both the acute and chronic stages of the SNL model average between 65–75 g, whereas SNL surgery induces a marked mechanical allodynia with thresholds of 20–30 g [52]. Mechanosensitivity was also measured using a paw compression test on the left hind paw while each rat was restrained in the recording system that was also used for vocalization measurements (see Section 4.6.2 “Emotional-affective responses”). For this behavioral assay, the rats were briefly anesthetized with isoflurane (2–3%; precision vaporizer, Harvard Apparatus) to allow for an easy positioning in a customized holding chamber that provided slight restraint but allowed hind limb access (U.S. Patent 7,213,538). Following recovery from anesthesia and a 30 min habituation to the recording chamber, hind limb withdrawal thresholds were measured using a calibrated forceps with a force transducer. The left hind paw was compressed with gradually increasing intensity until a reflex response was evoked, as described in our previous studies [51,54,112]. The withdrawal threshold was defined as the force required to evoke this reflex response and was calculated using the average value from three separate trials. Utilization of these two mechanosensitivity assays allows for the assessment of cutaneous (von Frey test) and additional deep tissue (paw compression test) components of mechanical hypersensitivity [191,192,193].

#### 4.6.2. Emotional-Affective Responses

Emotional-affective responses were evaluated by measuring audible (20 Hz–16 kHz) and ultrasonic (25 ± 4 kHz) vocalizations evoked by noxious test stimuli as described before [54,112,194]. The rats were briefly anesthetized with isoflurane (2–3%; precision vaporizer, Harvard Apparatus) to allow for a gentle and slightly restrained placement in the customized holding chamber that allows hind limb access (see Section 4.6.1 “Mechanosensitivity”). The rats were allowed to recover from anesthesia and habituate to the recording chamber for 30 min before beginning each experimental trial. Vocalizations were evoked by a brief (10 s) normally innocuous (100 g/6 mm^2^) or noxious (500 g/6 mm^2^) stimulus applied to the left hind paw using the calibrated forceps with a force transducer. Note that neuropathic rats show hypersensitivity (i.e., a decreased mechanical threshold) and therefore a lower stimulus intensity (normally innocuous) is sufficient to evoke vocalizations [54]. Vocalizations were automatically detected for 1 min using a full-spectrum microphone (max sampling rate: 384 kHz), and the total duration (in s) of the audible and ultrasonic components of vocalizations following the onset of the mechanical stimulus were analyzed using UltraVox 3.2 software (Noldus Information Technology). The duration (in s) of each individual call was summed at the end of each 1 min recording period to determine the total duration of the audible and ultrasonic vocalization components for each rat. 

#### 4.6.3. Anxiety-Like Behaviors

Anxiety-like behavior was evaluated in the open field test (OFT) and the elevated plus maze (EPM). The OFT was used to measure exploratory behavior within the peripheral and central zones of an open arena (70 cm × 70 cm) with acrylic walls (height of 45 cm). Rat movements were recorded for 15 min through a computerized video tracking and analysis system (EthoVision XT 11 software, Noldus Information Technology, Leesburg, VA, USA) as previously described [54,112,194]. The number of entries and the time spent in the center zone of the arena (35 cm × 35 cm) was calculated during the first 5 min of the experimental trial for each rat. Locomotor activity was measured as the total distance (in cm) that the rat traveled during the 15 min experimental trial. Avoidance of the center zone of the arena is interpreted as anxiety-like behavior [195,196]. The EPM contains two open arms and two closed arms (arm length, 50 cm; arm width, 10 cm; wall height, 40 cm) that are connected by a central zone (10 cm × 10 cm) at a height of 60 cm above the floor. At the beginning of each experimental trial, rats were placed in the center zone facing one open arm as described previously [52,100,194]. Similar to the OFT, rat exploratory movements in the EPM were recorded for 15 min through a computerized video tracking and analysis system (EthoVision XT 13 software, Noldus Information Technology). Open arm choice and open arm duration were calculated as the percentage of total entries and the percentage of total time spent, respectively, in the open arms during the first 5 min of each experimental trial. Avoidance of the open arms of the EPM is interpreted as anxiety-like behavior [197].

### 4.7. Statistical Analysis

Statistical significance was accepted at the level *p* < 0.05. All averaged values are presented as the means ± SEM. GraphPad Prism 9.5.1 software (Graph-Pad Software, San Diego, CA, USA) was used for all statistical analyses. For the analysis of normalized counts from RNA sequencing, qRT-PCR experiments, and behavioral experiments, a two-way ANOVA with Šidák’s post hoc tests was used for multiple comparisons [198].

## 5. Conclusions

The data may suggest that while Hmgb1 signaling mechanisms play a critical role in neuropathic pain-related amygdala function, this influence likely differs with respect to the brain hemisphere and the time course of neuropathic pain progression in both males and females. Hmgb1 was demonstrated to have a delayed upregulation in neuropathic pain that was specific to the right amygdala, which was reflected in the beneficial effects of silencing its expression following rather than preceding neuropathic pain induction. Hmgb1 in the amygdala may play a stronger role in the mechanosensory and emotional-affective components of pain than in associated anxiety-like behaviors. Hmgb1 inhibition in the amygdala may serve as a novel therapeutic strategy for chronic neuropathic pain relief in both sexes, though future investigation into the cell type-specific mechanisms of neuroimmune signaling within the amygdala in a neuropathic pain state is warranted. The present study provides support for the exploration of therapeutic strategies that target neuron–glia interactions across all stages of neuropathic pain development.

## Data Availability

The original contributions presented in the study are included in the article. Further inquiries can be directed to the corresponding author.

## Supplementary Materials

The supporting information can be downloaded at: https://www.mdpi.com/article/10.3390/ijms241511944/s1. Reference [199] is cited in the supplementary materials.
