# Supplementary material for: Hmgb1 Silencing in the Amygdala Inhibits Pain-Related Behaviors in a Rat Model of Neuropathic Pain
Source: Int J Mol Sci. 2023 Jul 26;24(15):11944. doi: 10.3390/ijms241511944 (PMC10418916; doi:10.3390/ijms241511944)
Supplement: Supplementary file 1 [file ijms-24-11944-s001.zip › ijms-2426276-supplementary.pdf]

A Expression of Hmgb1 on neurons in the CeA

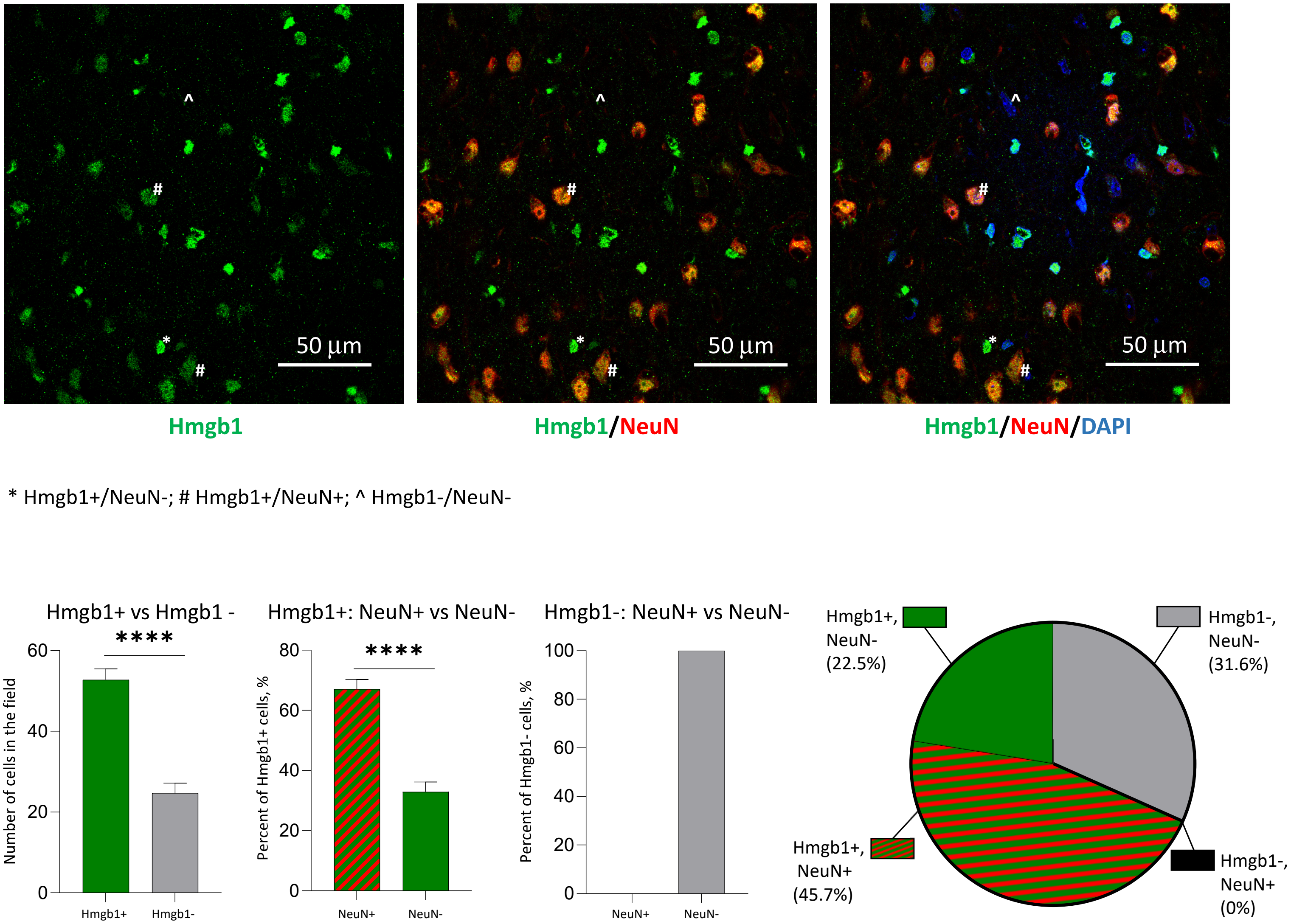

B Expression of Hmgb1 on CRF neurons in the CeA

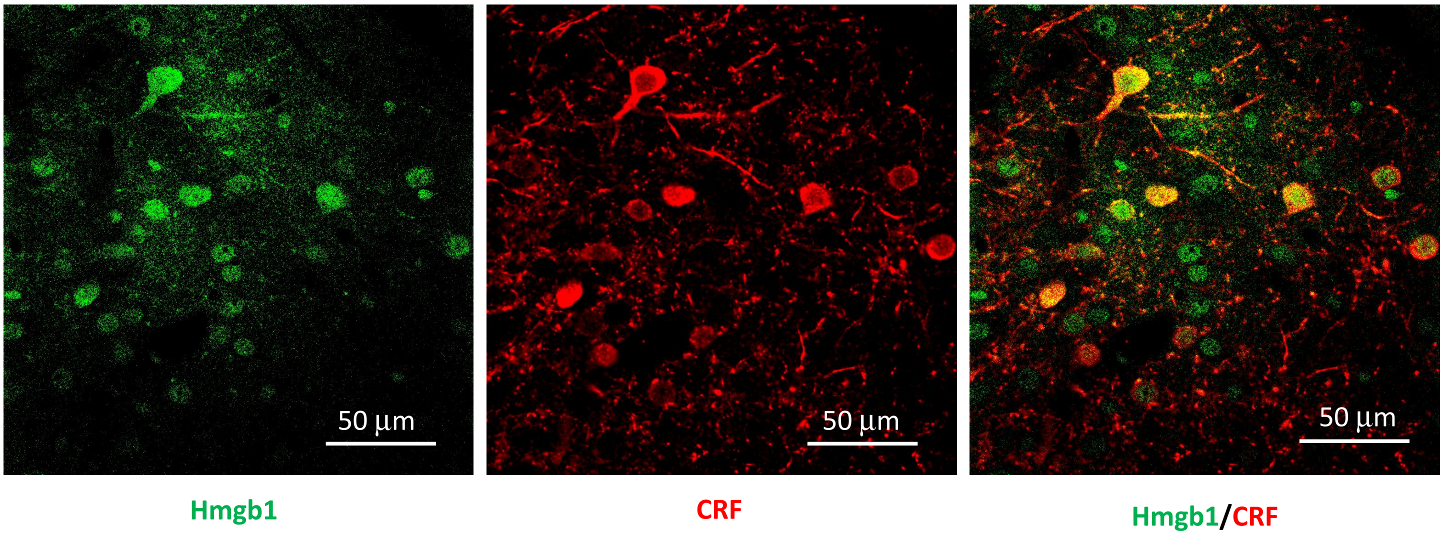

Supplementary Figure 1

Figure S1: Neuronal expression of Hmgb1 protein in the central nucleus of the amygdala. For immunohistochemical studies, male rats were deeply anesthetized (4% isoflurane; precision vaporizer, Harvard Apparatus) and transcardially perfused with 0.1 M phosphate-buffered saline (PBS), followed by 4% paraformaldehyde (PFA) in PBS. Brains were extracted and fixed at 4°C overnight in 4% PFA, then cryoprotected in a 30% sucrose solution for 48 hours and embedded in optimal cutting temperature (OCT) compound. Coronal 40 μm sections were prepared using a cryostat (HM525NX, Epredia). Sections were permeabilized in 0.3% Triton in PBS (PBST) for 10 minutes, blocked in 5% Normal Goat Serum (NGS) in PBS for 1 hour, and incubated with the primary antibodies (α-HMGB1, Mouse, R&D Systems, #MAB1690, 1:100 dilution; α-NeuN (neuron-specific nuclear protein), Guinea Pig, Millipore, ABN90, 1:1000 dilution) with 5% NGS in PBS overnight. The next day, sections were washed 3x5 minutes in PBST and then incubated in secondary antibodies (Goat-anti-Mouse Alexa 488, A11029, and Goat-anti-Guinea Pig Alexa 647, A21450; Thermo Fisher) for 2 hours at room temperature. After washing 3x5 minutes in PBS, sections were mounted in ProLong antifade media (P36935, Thermo Fisher). For the quantification of Hmgb1+ cells, sections were imaged using a confocal microscope (Olympus FV3000) with 60X oil immersion objective in three channels: DAPI, Hmgb1, and NeuN. Image analysis was performed with ImageJ software (v1.53f51, NIH). All nuclei were traced in the DAPI channel and saved in ROI Manager to count the number of cells in the field. Afterward, areas from ROI Manager were transposed to a channel with Hmgb1 signal to count Hmgb1+ cells. In order to calculate the percentage of NeuN+ cells in the population of Hmgb1+ cells, areas with Hmgb1 signal from ROI Manager were transposed to the NeuN channel. **(A)** Confocal images and quantification of Hmgb1 expression in the CeA. Hmgb1+ cells make up 68.2% of total cells. Of Hmgb1+ cells, the majority (67%) co-localize with NeuN+ cells in the CeA. Importantly, all NeuN+ cells are Hmgb1+ and they make up 45.7% of all CeA cells. ****, $P < 0.0001$, unpaired t-test. Error bars show means ± SEM. Scale bar, 50 μm. **(B)** Confocal images of Hmgb1 and CRF expression in the CeA. Genetically modified Crh-Cre rats that express Cre-dependent recombinase exclusively in CRF-containing neurons [199] were used to visualize CRF neurons, as described previously in our laboratory [50,51]. Confocal image of mCherry fluorescence in a brain slice from a Crh-Cre rat shows neurons expressing CRF in the CeA 4-5 weeks after rAAV5/EF1a-DIO-mCherry (1 μL, 10^{12} units / 100 μL; vector core facility, University of North Carolina) injection into the CeA. Hmgb1 is co-expressed with CRF neurons in the CeA. Scale bar, 50 μm.
